# Unusual Cause of Acute Urinary Retention in Young Male Patient: Primary Synovial Sarcoma of Prostate—A Case Report

**DOI:** 10.1055/s-0042-1758052

**Published:** 2022-12-02

**Authors:** Santhoshkumar Bandegudda, Rakesh Sharma Manilal, Ashwin Giridhar, B. Vishal Rao

**Affiliations:** 1 Department of Surgical Oncology, Basavatarakam Indo-American Cancer Hospital and Research Institute, Hyderabad, Telangana, India; 2 Division of Uro-Oncology, Department of Surgical Oncology, Basavatarakam Indo-American Cancer Hospital and Research Institute, Hyderabad, Telangana, India; 3 Department of Pathology, Basavatarakam Indo-American Cancer Hospital and Research Institute, Hyderabad, Telangana, India

**Keywords:** primary synovial sarcoma, prostate sarcoma, prostate synovial sarcoma, sarcoma, synovial sarcoma

## Abstract

**Introduction** Primary synovial sarcoma (SS) of the prostate is the rarest variety of prostate sarcoma. The first documented and confirmed case of SS of the prostate was published by Iwasaki et al in the year 1999; since then, only a few cases of primary SS of the prostate have been published in English literature.

**Case Report** We report a unique case of primary SS in a young patient who presented with acute urinary retention and underwent emergency suprapubic catheterization, and on evaluation was diagnosed with primary SS of the prostate. Patient was managed with radical cystoprostatectomy and resection of the anterior wall of rectum infiltrated by the tumor with bilateral pelvic lymph node dissection and adjuvant chemotherapy. Patient died after 2 months of surgery.

**Conclusion** Primary SS of the prostate is a rare disease and important clinical entity to be included in differential diagnosis of acute urinary retention in young patients. It is associated with high local recurrence and poor prognosis, which warrants multidisciplinary approach of treatment.

Primary soft tissue tumor of the prostate is a rare entity that arises from the mesenchymal part of the prostate. It accounts for 0.7% of all primary prostate malignancies. The predominant histological subtypes of prostate sarcoma are leiomyosarcoma in adults and rhabdomyosarcoma in children, 1 whereas synovial sarcoma (SS) of the prostate is the rarest variety of primary prostate sarcoma. Lower urinary tract symptoms (LUTS) are the commonest presentation in this subset of patients. However, the diagnosis is routinely established by digital rectal examination, histopathology, imaging, and serum prostate specific antigen (PSA) levels. Preliminary evaluation of serum PSA is normal in majority of the cases and helps to differentiate prostate sarcoma from other causes of enlarged prostate including prostate hyperplasia and carcinoma. Unlike any other sarcoma, surgery is the standard of care with the role of chemotherapy and radiotherapy remaining uncertain. There is a high local recurrence rate and poor prognosis in prostate sarcoma. The first documented and confirmed case of SS of the prostate was published by Iwasaki et al in the year 1999. 2 Since then, only a few cases of primary SS of the prostate have been published in English literature. We are reporting a unique case of primary SS treated in a tertiary cancer center.

## Case Report

**Fig. 1:**
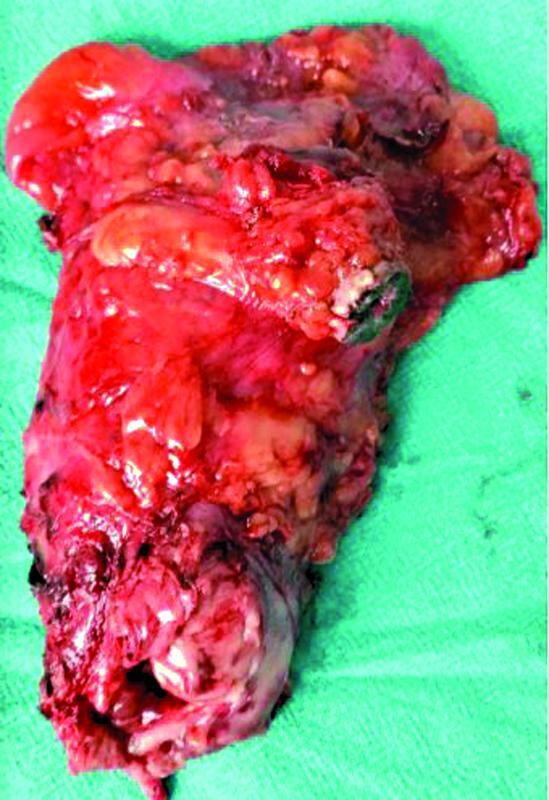
Gross specimen of cystoprostatectomy with en bloc excision of suprapubic catheter tract.

**Fig. 2:**
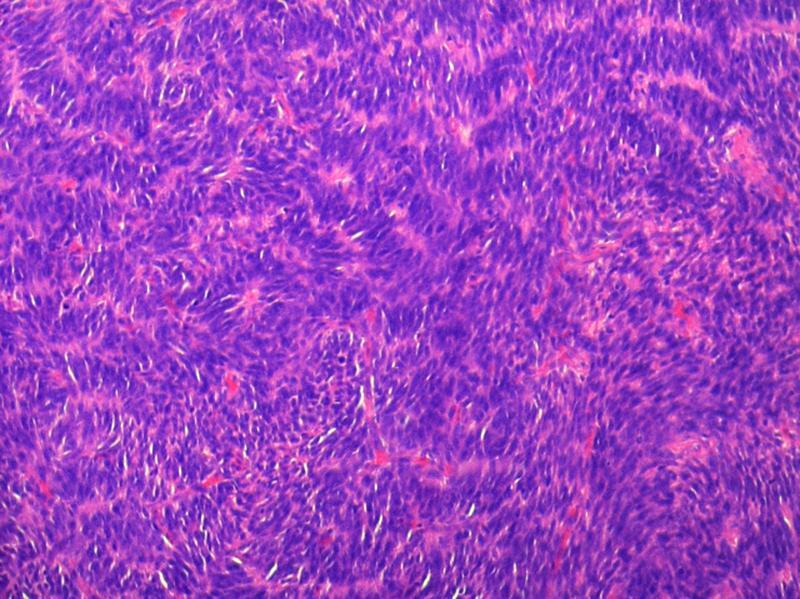
Spindle cells arranged in short fascicles with moderate atypia.

**Fig. 3:**
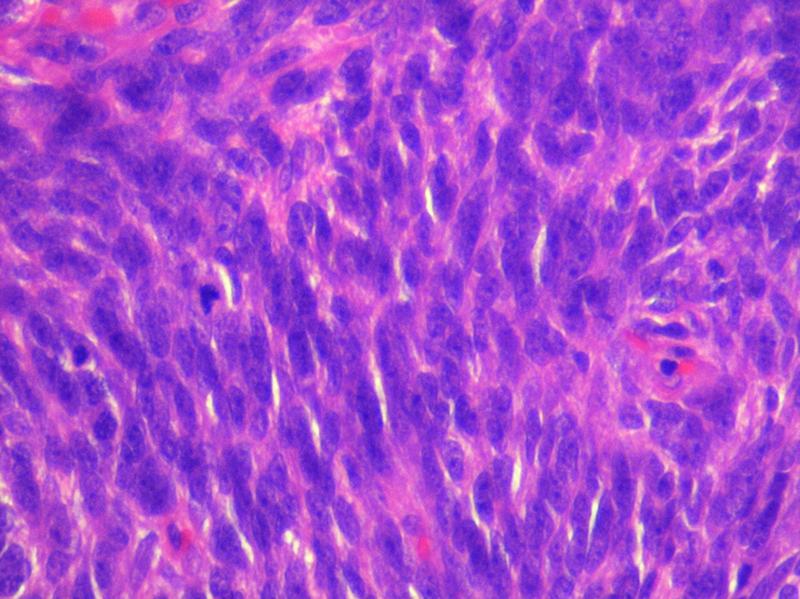
Spindle cells with atypical mitosis.

**Fig. 4:**
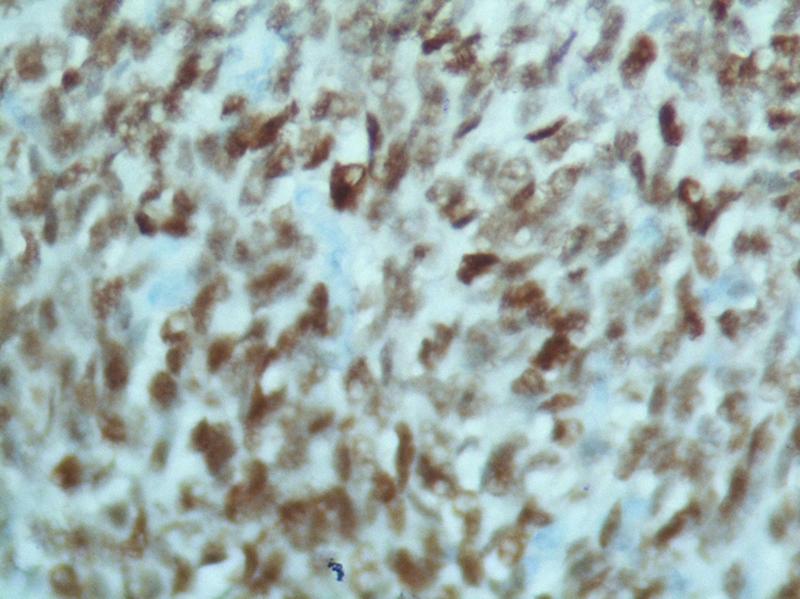
Positive stain of tumor cells for TLE1 transducine-like Enhancer 1 (TLE-1).

**Fig. 5:**
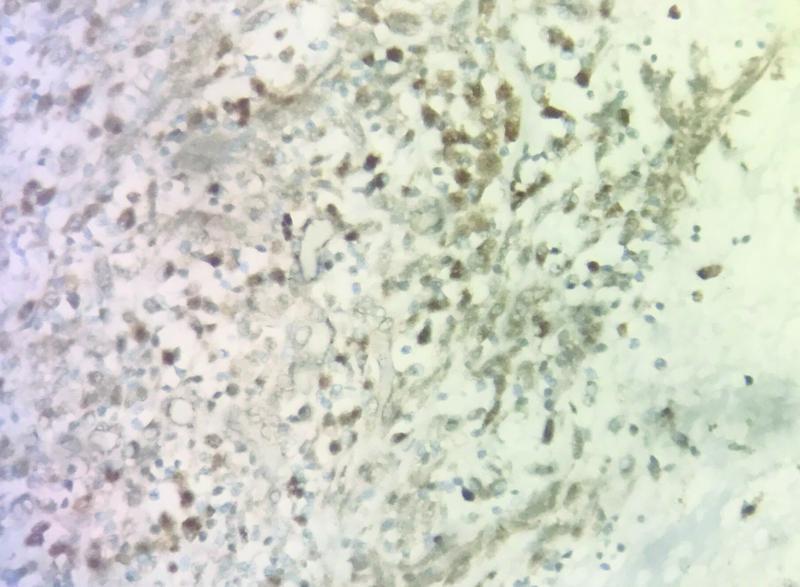
Positive stain of tumor cells for SS18.

**Fig. 6:**
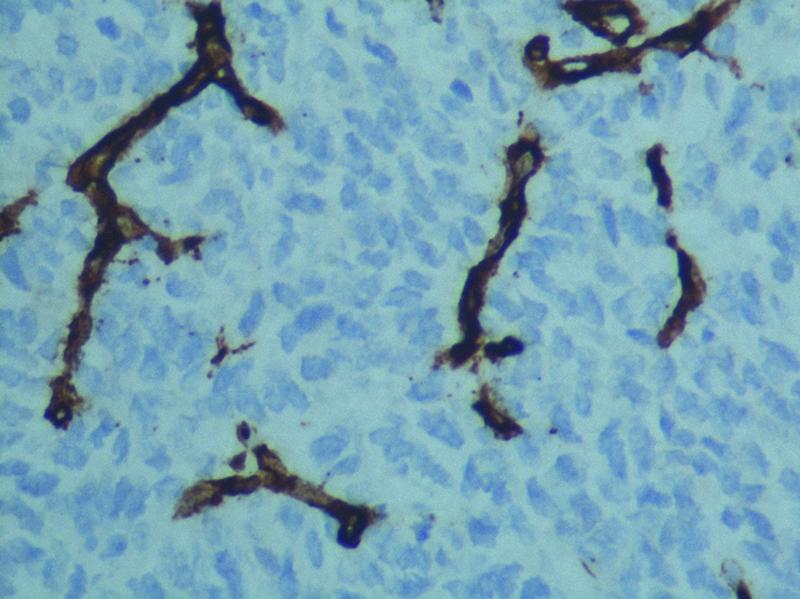
Negative stain of tumor cells for CD34. CD 34, cluster of differentiation 34.

**Fig. 7:**
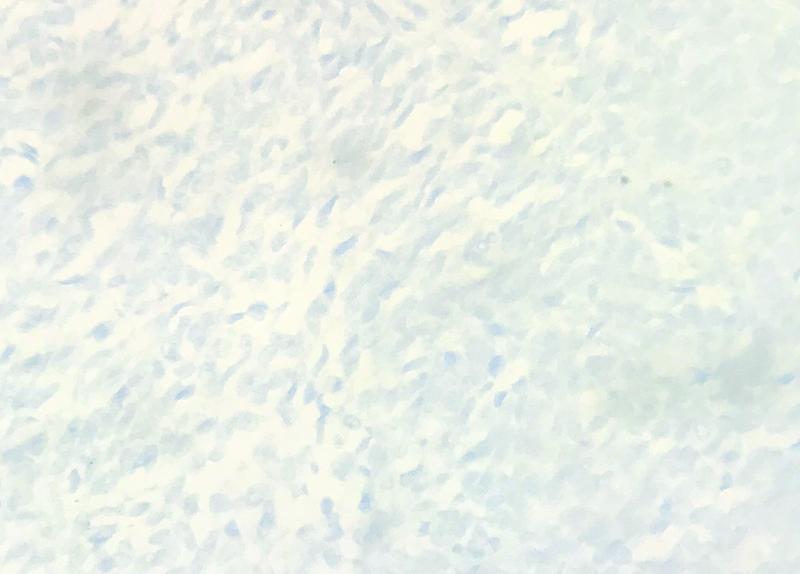
Negative stain of tumor cells for PR, progesterone receptor.

A 35-year-old male, farmer, belonging to lower socioeconomic status, with a history of smoking cigarettes for the past 10 years and no comorbidity, presented to the emergency with acute urinary retention with no other significant complaints. There was no history of similar episodes in past, nor there was any previous history of periurethral catheterization. None of the family members of patient had cancer or similar complaints. Due to failed periurethral catheterization, suprapubic catheterization was done. On further evaluation, transrectal ultrasound revealed diffusely enlarged prostate measuring 44 × 48 × 49 mm, with a volume of 55 cc, and with altered echotexture and lateralization toward left. Magnetic resonance imaging (MRI) was suggestive of a lesion showing diffusion restriction with postcontrast heterogeneous enhancement and minimal extraprostatic extension on the left and posteriorly to the base of the bladder. The patient received preoperative antibiotic treatment for urinary tract infection. Transrectal biopsy was done, which was suggestive of spindle cell tumor. Patient was treated in the Department of Surgical Oncology, Division of Uro-Oncology, Basavatarakam Indo-American Cancer Hospital and Research Institute, Hyderabad, India. Preoperative fitness for surgery was assessed and open radical cystoprostatectomy with resection of the anterior wall of rectum infiltrated by the tumor and the suprapubic tract with bilateral pelvic lymph node dissection with an ileal conduit was done under general anesthesia ( Fig. 1 ) by senior most uro-oncologist. Patient had an uneventful postoperative recovery and was discharged on postoperative day 5. Histopathology was suggestive of neoplastic spindle cells with moderate cytoplasm and pleomorphic oval to spindle vesicular nuclei with dispersed chromatin; few cells had prominent nucleoli. The cells were arranged in bundles and infiltrating fascicles with extensive areas of necrosis and hemorrhage noted. There was no lymphovascular or perineural invasion or extraprostatic fat extension; margins were negative and there was no lymph node metastasis ( Figs. 2 , 3 ). On the basis of hematoxylin and eosin, a differential diagnosis of primary prostate stromal sarcoma, primary SS, and solitary fibrous tumor of prostate was considered. Further immunohistochemistry (IHC) was performed, which was positive for transducine-like Enhancer 1 (TLE-1) ( Fig. 4 ), cluster of differentiation 99 (CD99), friend leukemia integration 1 (FLI), and B cell lymphoma 2 (BCL-2); focal positive for SS18-SSX ( Fig. 5 ); negative for epithelial membrane antigen (EMA), cluster of differentiation 34 (CD34) ( Fig. 6 ), progesterone receptor (PR) ( Fig. 7 ), and synaptophysin; and integrase interactor (INI 1) was retained, which were suggestive of primary SS of prostate. The case was discussed in a multidisciplinary tumor board and patient was given one cycle of adjuvant chemotherapy—ifosfamide and adriamycin. The patient died after receiving one cycle of chemotherapy, cause unknown. Written informed consent was obtained from the patient during hospital admission for publication of this case report and accompanying images. A copy of the written consent is available for review by the Editor-in-Chief of this journal on request.

## Discussion

SS accounts for approximately 10 to 20% of all soft tissue tumors. It is typically seen among young adults with a median age of 35 years and ranging from 5 to 85 years. 3 SS arises from a multipotent mesenchymal stem cell resulting from a deregulation of self-renewal and differentiation capacities led by t(X;18)(p11;q11) chromosomal translocation, which leads to the formation of SS18-SSX fusion gene. 4 Based on histology, SS is classified into four broad categories according to the proportion of epithelial to spindle cells: (1) biphasic, (2) monophasic fibrous type, (3) monophasic epithelial type, and (4) poorly differentiated type. There is a strong correlation between SYT-SSX fusion transcript and histology pattern. SYT-SSX1 is associated with biphasic form and SYT-SSX2 fusion is associated with monophasic histology. 5 Most of the SS arises from extremities. However, few are seen in locations like heart, kidney, retroperitoneum, lung, and cerebellum. 6 In patients with localized SS, surgery remains the treatment of choice, whereas the defined role of chemotherapy or radiotherapy to improve survival remains elusive. 7

SS of the prostate is the rarest entity. To our knowledge, only 12 cases of primary prostate SS are reported to date ( Table 1 ). Iwasaki et al (1999) published a case of PSS in a 37-year-old patient having monophasic tumor cells positive for vimentin, focally positive for epithelial membrane antigen, and negative for keratin, S-100, desmin, muscle-specific actin, and α-smooth muscle actin. This was the first case to demonstrate and confirm translocation of t(X;18)(p11;q11) by fluorescence in situ hybridization (FISH) in a case of PSS. 2

**Table 1:** Clinical details of published cases of primary synovial sarcoma prostate

Author	Patient age (years)	PSA level (ng/mL)	Symptoms	Histopathology	Treatment	Follow-up
Iwasaki et al (1999) 2	37	–	–	Monophasic tumor cells positive for vimentin, focal positive for EMA, and negative for keratin, S-100, desmin, MSA, and ASMA; t(X;18)(p11;q11) conﬁrmed by FISH	–	–
Fritsch et al (2000) 11	34	–	Prostatism	Round cell and spindle cell; vimentin, myosin, and actin were positive; desmin, PSA, and PSAP were negative; S100, EMA, myoglobin, and cytokeratin were equivocal	Radial cystoprostatectomy with en bloc resection of rectum	Patient died of bilateral lung metastases after 3 years
Shirakawa et al (2003) 14	52	0.9	Urinary retention	Monophasic synovial sarcoma arising from the prostatic fascia; similar IHC positive for vimentin and negative for ASMA, desmin, and S-100; t(X;18)(p11.2;q11.2) was conﬁrmed by FISH	Retropubic radical prostatectomy and adjuvant chemotherapy with ifosfamide and doxorubicin	6-month follow-up with no recurrence
Williams et al (2004) 9	63	0.5	LUTS	–	Preoperative EBRT for 5 weeks, 50.4 Gy-10% decrease in tumor, and anterior pelvis exenteration with en bloc penectomy and pubectomy	–
Pan CC (2006) 10	44	2.91	LUTS	Monophasic spindle cell sarcoma; positive for BCL-2; focally positive for CD99; negative for EMA, actin, desmin, HHF35, and S-100; confirmed by RT-PCR type 2 SYT-SSX	Radical prostatectomy and adjuvant ifosfamide chemotherapy	–
Jun et al (2008) 13	46(1)	0.34	Dysuria	Monophasic spindle cell; positive for vimentin, BCL-2, CD99, and E-cadherin; focally positive for cytokeratin; negative for PSA, S-100, CD34, CD117, actin, desmin, and calretinin; trans location confirmed by RT-PCR	Radical prostatectomy with negative surgical margins	8-month follow-up with no recurrence or metastasis
–	44(2)	1.18	Dysuria and painful micturition	Monophasic spindle cell; positive for vimentin, BCL-2, CD99, and E-cadherin; focally positive for cytokeratin; negative for PSA, S-100, CD34, CD117, actin, desmin, and calretinin; trans location confirmed by RT-PCR	Incomplete resection of tumor	Recurrence of tumor in pelvis and costosternal metastasis; died due to pulmonary metastasis after 8 months
Dhabalia et al (2009) 15	25	1.7	Acute urinary retention	IHC positive for vimentin and BCL-2 and focally positive for cytokeratin; S-100 and calponin were negative	Total pelvic exenteration with negative margins	NA
Zhang et al (2014) 8	22	1.2	LUTS	IHC positive for vimentin and CD99 and negative for ASMA, desmin, and S-100; SYT-SSX fusion confirmed by RT-PCR	No treatment received	Died after 3 months due to lung metastasis
Olivetti et al (2015) 16	46	1.03	Acute urinary retention	IHC suggestive of pankeratin expression confined to scattered, individual spindle cells, and diffuse expression of CD56, CD99, and BCL-2; S-100, protein, muscle actin, desmin, and CD34 were negative; FISH for t(X;18)(p11q11) was positive	Debulking surgery with postoperative chemotherapy with ifosfamide and epirubicin	Persistent disease
Maleki et al (2017) 17	38	0.58	Prostatitis	Immunoreactivity for cytokeratins AE1:3 and CAM 5.2; strongly positive for vimentin and BCL-2 and focally positive for calretinin and CK7; chromogranin, synaptophysin, S-100, SMA, desmin, CD31, CD34, CK20, WT-1, and AFP were negative; CD99 (O-13) was noncontributory; FISH for t(X;18)(p11q11) was positive	Robotic-assisted laparoscopic radical prostatectomy and concomitant chemoradiation	Died after 2 years due to pulmonary metastasis
Hou et al (2022) 12	42	0.81	Dysuria	IHC positive for vimentin, CD99, BCL-2, and SS18-SSX and negative for S-100, CD34, CD117, SMA, desmin, WT-1, calretinin, EMA, CK, CAM 5.2, CK7, CK19, MyoD1, and myogenin; FISH for t(X;18)(p11q11) was positive	Palliative treatment	Died after 18 months of diagnosis

Abbreviations: AE, Cytokeratine AE1:3; AFP, Alpha-fetoprotein; ASMA, anti smooth muscle antibody; BCL-2, B-cell lymphoma 2; CAM; CD, cluster of differentiation; CK, cytokeratin; EBRT, external beam radiotherapy; EMA, Epithelial membrane antigen; FISH, fluorescence in-situ hybridization; HHF35, muscle specific antigen; IHC, immunohistochemistry; LUTS, lower urinary tract symptoms; MSA, muscle specific antigen; NA, not available; PSA, prostate specific antigen; PSAP; RT-PCR, reverse transcriptase polymerase chain reaction; SS18, synovial sarcoma; SYT-SSX; WT-1, wilms tumor 1.

The youngest case was reported in a 22-year-old 8 patient, and others are seen in patients with an age of 25 to 63 years. The presenting symptoms are LUTS, 8 9 10 11 dysuria, 12 13 and acute urinary retention. 14 15 16 Some may also have features of prostatitis. 16 Unlike elevated serum PSA in prostate carcinoma, PSA level will be in a normal range in prostate SS. The median size of the tumor on presentation is 5 cm with imaging features similar to any other sarcoma, including heterogeneous enhancement and diffusion-weighted restriction on contrast MRI. Monophasic spindle cell type is the commonest type, which is seen in most of the cases and also in our patients. IHC positive for vimentin, CD99, and BCL-2, focally positive for cytokeratin, and negative for desmin, actin, and S-100 suggests SS. Presence of t(X;18)(p11;q11) chromosomal translocation by reverse transcriptase polymerase chain reaction or FISH confirms the diagnosis. 2 3 4 5 6 7 8 9 10 11 12 13 14 15 16 17

Surgery is the primary modality of treatment for prostate SS with en bloc resection of involved organs to obtain negative margin. However, the role of debulking surgery is unknown. 16 Neoadjuvant radiation tried in one case showed 10% reduction in size. 10 The role of adjuvant chemotherapy is uncertain. Considering the high-grade stage of tumor and poor prognosis of disease in few patients, adjuvant chemotherapy was given. 9 11 16 17 Our patient underwent radical cystoprostatectomy with resection of the anterior wall of rectum infiltrated by the tumor with bilateral pelvic lymph node dissection with ileal conduit and received one cycle of adjuvant chemotherapy. The most common site of metastasis is lung 9 12 15 17 and is associated with poor prognosis, indicating need of regular follow-up with clinical examination and imaging of lung. 8 12 15 17

## Conclusion

Primary SS of the prostate is a rare disease associated with poor prognosis. It should be considered as a differential diagnosis in young patients presenting with acute urinary retention with enlarged prostate and normal PSA level. Early detection and radical surgery with multidisciplinary approach may provide a good outcome.
